# Tumor Hypoxia Regulates Immune Escape/Invasion: Influence on Angiogenesis and Potential Impact of Hypoxic Biomarkers on Cancer Therapies

**DOI:** 10.3389/fimmu.2020.613114

**Published:** 2021-01-20

**Authors:** Raefa Abou Khouzam, Klaudia Brodaczewska, Aleksandra Filipiak, Nagwa Ahmed Zeinelabdin, Stephanie Buart, Cezary Szczylik, Claudine Kieda, Salem Chouaib

**Affiliations:** ^1^ Thumbay Research Institute for Precision Medicine, Gulf Medical University, Ajman, United Arab Emirates; ^2^ Laboratory of Molecular Oncology and Innovative Therapies, Military Institute of Medicine, Warsaw, Poland; ^3^ Postgraduate School of Molecular Medicine, Medical University of Warsaw, Warsaw, Poland; ^4^ INSERM UMR 1186, Integrative Tumor Immunology and Genetic Oncology, Gustave Roussy, EPHE, Faulty. De médecine Univ. Paris-Sud, University Paris-Saclay, Villejuif, France; ^5^ Centre of Postgraduate Medical Education, Department of Oncology, European Health Centre, Otwock, Warsaw, Poland; ^6^ Centre for Molecular Biophysics, UPR CNRS 4301, Orléans, France

**Keywords:** microenvironment, angiogenesis, hypoxia, vessel, normalization, tumor suppressors, signatures

## Abstract

The environmental and metabolic pressures in the tumor microenvironment (TME) play a key role in molding tumor development by impacting the stromal and immune cell fractions, TME composition and activation. Hypoxia triggers a cascade of events that promote tumor growth, enhance resistance to the anti-tumor immune response and instigate tumor angiogenesis. During growth, the developing angiogenesis is pathological and gives rise to a haphazardly shaped and leaky tumor vasculature with abnormal properties. Accordingly, aberrantly vascularized TME induces immunosuppression and maintains a continuous hypoxic state. Normalizing the tumor vasculature to restore its vascular integrity, should hence enhance tumor perfusion, relieving hypoxia, and reshaping anti-tumor immunity. Emerging vascular normalization strategies have a great potential in achieving a stable normalization, resulting in mature and functional blood vessels that alleviate tumor hypoxia. Biomarkers enabling the detection and monitoring of tumor hypoxia could be highly advantageous in aiding the translation of novel normalization strategies to clinical application, alone, or in combination with other treatment modalities, such as immunotherapy.

## Introduction

The tumor microenvironment is a complex system, playing an important role in tumor development and progression. Besides cellular stromal components, extracellular matrix fibers, cytokines, and other metabolic mediators are also involved (1, 2). Among the microenvironmental factors that play a dominant role in neoplasia, hypoxia is believed to be one of the most relevant in the neoplastic response of tumor cells (3). It is widely appreciated that the majority of malignancies create a hostile hypoxic microenvironment that can hamper cell-mediated immunity and dampen the efficacy of the immune response (4, 5). Hypoxia, as an integral component of the tumor microenvironment and especially of the pathologically vascularized zones inside solid tumors contributes to immune tolerance of tumor cells by impeding the homing of immunocompetent cells into tumors and inhibiting their antitumor efficacy (6).

**Figure 1:**
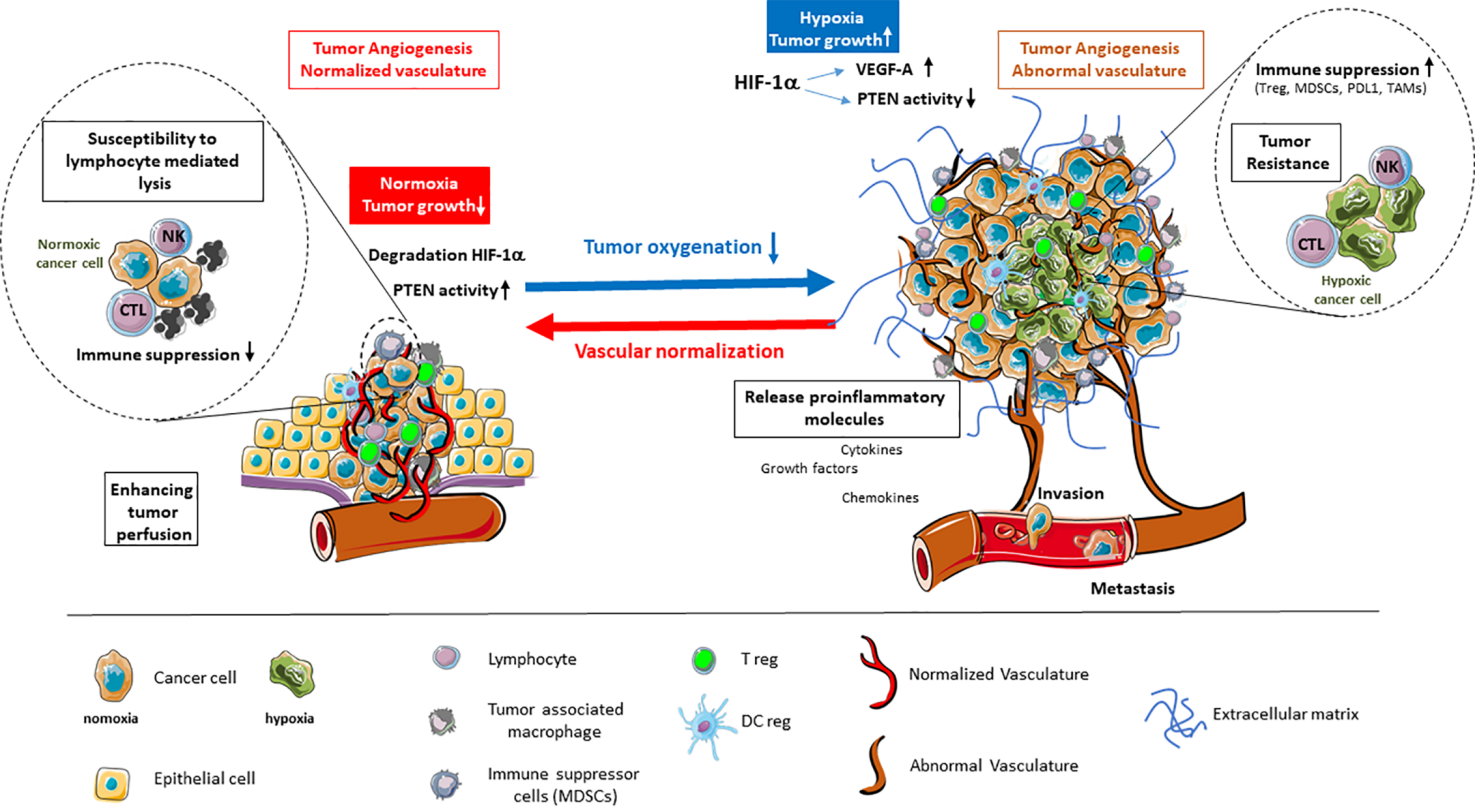
Alleviation of tumor hypoxia by vascular normalization reprograms the tumor microenvironment. Hypoxia in the tumor microenvironment promotes tumor angiogenesis and abnormal vascularization through the activation of HIF-1α and downstream effector genes, such as VEGF-A, while inhibiting the tumor suppressor PTEN. This is coupled by enhanced immune suppression, the release of proinflammatory molecules, as well as the promotion of invasion and metastasis. The alleviation of tumor hypoxia through vascular normalization enhances tumor perfusion, leading to the degradation of HIF-1α and restoration of PTEN activity. The reestablishment of normal oxygenation additionally counteracts tumor growth through the revitalization of the immune response.

It is established that the endothelial response to tumor hypoxia signaling is the angiogenic switch involving HIF-1α stabilization and transcription, along with production of VEGFs, angiopoietin 2, IL-8, and other factors. As antiangiogenic treatments have shown that destruction of angiogenesis leads to deep hypoxia and induces tumor resistance (7), efforts are focusing on rendering the tumor vessels functional to help treat the tumor (8, 9). By normalizing vessels, the challenge is to alleviate hypoxia to counteract most deleterious effects related to tumor microenvironment (**Figure 1**).

Normalization of vessels is a strategy to: first allow a new delivery of red blood cells, thus balancing the oxygen partial pressure inside the capillaries, and reducing the activity of the enzymatic pathways that sense the pO_2_ low level; second, to down regulate the hypoxia-activated molecular cascades that make the vessels chaotic and permeable, thus reducing the ability to maintain a flow in the capillaries (10). While normalized, vessels allow the blood borne molecules and cells to reach the internal part of the tumor site. As such, they permit the use of therapeutic strategies among which immunotherapies (11). Moreover, the consequences of such a normalization on the availability of O_2_ are deeply influencing the reactional balance by acting on soluble factors, regulating the extracellular matrix molecules and their activity, and impacting the metabolism and phenotype of the cells inside the microenvironment of the tumor cells (TME), as well as exocrine and paracrine signals.

Regulation of angiogenesis and recovery from its pathologic state is the challenge to which tumor suppressor molecules by their activation, may bring a considerable mean to progress. In the angiogenic process the PTEN molecule is a key regulator which orchestrates the structure of the vessels (12–14). This places endothelial PTEN as a key target for normalization strategies.

Given the negative outcome associated with tumor hypoxia, a major goal has been recently to identify hypoxic tumors through a number of different approaches. In this regard, several hypoxia signatures have been reported and used to classify patient tumors into high-hypoxia and low-hypoxia, based on a hypoxia score generated from the expression of the signature genes in tumor samples (15–18). This would presumably enable the clinical development of personalized, hypoxia-based therapies, which will, ultimately, improve outcomes in particular in cancer immunotherapy.

We will discuss available data on the relationship between hypoxia- and immune-related genes, how they are in most cases linking tumor hypoxia with an immunosuppressed state in cancer patients and more importantly have the potential to generate prognostic information. Clearly tumor hypoxia is a key component of TME interfering with the angiogenic axis, the remodeling of vessel architecture, function and immune reaction. In this respect, hypoxia signatures should be further considered as potential biomarkers that may lead to significant progress in the field of cancer immunotherapy.

## Hypoxia in the Tumor Microenvironment Promotes Angiogenesis

In the context of solid tumors, microenvironment is characterized by a network of cancer cells, resident, recruited stromal and immune cells, as well as non-cellular components of the extracellular matrix (ECM) (1, 19). The crosstalk occurring among these various elements, through direct contacts and through an array of soluble factors, nurtures a dynamic TME in which altered tumor and host cell phenotypes are acquired (1, 2).

The early stages of tumor development are characterized by highly proliferating tumor cells which are aided in their growth and epithelial-mesenchymal transition by cancer associated fibroblasts (CAFs). These cells arise from resident fibroblasts or from other progenitor cells, including smooth muscle cells, pericytes, and bone marrow-derived mesenchymal cells (20–22). The endothelial cell and pericyte populations in the TME are required for the switch of a tumor mass from an avascular growth phase to an angiogenic phase to support the growing tumor demands for oxygen and nutrients (23, 24). The resulting tumor vasculature has been described as haphazardly shaped with various degrees of leakiness and irregularities compared to normal surrounding vasculature (25). Regarding immune cells, the degree of tumor infiltration and the type of immune cells, being tumor promoting, such as tumor associated macrophages and myeloid derived stromal cells, or tumor inhibiting, such as NK cells and cytotoxic T cells, varies greatly (26). Indeed, the environmental and metabolic pressures in the TME have a key role in molding tumor development by impacting the nature of stromal and immune cell fractions, TME composition and activation state (2, 23, 27). In addition, non-cellular elements including components of the ECM, pH, and pO_2_ are of key importance in cancer development (28).

### Hypoxia in the Tumor Microenvironment Repurposes Tumor Cells, Instigating Angiogenesis

Hypoxia has been reported to exist in most solid tumors, manifesting when the diffusion of oxygen from surrounding vasculature is no longer adequately supporting the hyperproliferating tumor cells (29, 30). To sustain their energy, tumor cells need aerobic glycolysis, switching from metabolizing glucose through oxidative phosphorylation, to metabolizing it through glycolysis (Warburg effect) (31, 32). This process releases byproducts, such as lactic acid and carbonic acid, which if left unchecked would decrease intracellular pH resulting in acidosis (33). Hypoxia promotes transcriptional reprogramming of tumor cells with the aim of surpassing the detrimental environment of low oxygen and low pH levels. Such reprogramming is primarily executed by hypoxia inducible factors (HIFs) family of transcription factors. HIFs function as heterodimers composed of an oxygen-sensitive α-subunit (HIF-α) and a constitutive β-subunit (HIF-β). HIF-α exists in three isoforms, namely HIF-1α, HIF-2α, and HIF-3α, among which HIF-1α is at the forefront of the cellular response to hypoxia (30, 34–36).

With respect to angiogenesis, HIFs have been described as master regulators of this process (35). In particular, HIF-1 induces the expression of a range of pro-angiogenic genes, including VEGF (vascular endothelial growth factor), PGF (placenta growth factor), ANGPT-2 (angiopoietin-2), CXCL-12 (chemokine C-X-C motif ligand12), also referred to as SDF-1 (stromal-derived factor-1) and PDGF-B (platelet-derived growth factor-B). These factors are critical for facilitating the angiogenic switch in tumors upon binding their respective receptors on the surface of endothelial cells, pericytes, and vascular smooth muscle cells (35–38).

The most potent hypoxia pro-angiogenic factor is VEGF-A (36, 39). VEGF-A, -B, -C, -D, and PGF, are secreted and act on endothelial cells by binding VEGFR (VEGF receptor)-1 and -2 (38). This activates pathways that promote endothelial cell proliferation and survival, such as the ERK (extracellular regulated kinase) and PI3K (phosphatidylinositol 3-kinase)/AKT (protein kinase B) pathways (40); endothelial cell migration, like Rho GTPases (41); and ECM degradation for invasion and sprouting *via* expression of MT-MMP (membrane type matrix metalloproteinase) MMP-2, MMP-9, and urokinase plasminogen activator (40, 42). In addition, VEGF-A plays a key role in vascular permeability that is required for normal tissue homeostasis (43). Vascular hyper-permeability, due to the increased interstitial pressure in the tumor, enhances extravasation of tumor cells into the blood circulation and their metastasis (44). Indeed, while physiological angiogenesis gives rise to functional vessels, those arising in tumors are abnormal in structure and function, leading to inadequate tumor perfusion (35, 38, 45).

### Pseudohypoxia and VHL

Von Hippel-Lindau (pVHL) is an important tumor suppressor, which operates in the cell as a complex in which components are bound directly or indirectly to the pVHL (46–48). One of the most important roles of pVHL as E3 ubiquitin ligase is its targeted binding to HIF-1α when prolyl residues 402 and 564 are hydroxylated for oxygen-dependent proteolysis (49–51). In cancer, mutations of pVHL are frequently observed (52). Mutated or lost *VHL* gene, prevents participation of pVHL in the E3 ubiquitin ligase complex and interaction with its HIF-1α substrate, allowing stabilization of HIF-1α even in physiological levels of oxygen (53). This phenomenon is called “pseudohypoxia” i.e. induction of similar molecular mechanisms by VHL mutation as during exposure to non-physiological, low pO_2_ (described in previous section). Forty-six to 82% of sporadic cases of RCC are characterized by VHL mutations that result in the dysregulation of HIF-1 downstream genes, like VEGFs, PDGF, and TGFα and high angiogenic activity in these tumors (52). RCC patients with either *VHL* mutation (54) or high VEGF-A level (55) are characterized by poor prognosis. Indeed, similar to hypoxia, pseudohypoxia, exacerbates cancer progression and maintains a deep control on the TME, characterized by a strong immunosuppression (56) and a strong disturbance of the cell microenvironmental conditions.

### Hypoxia and PTEN-Mediated Regulation of Angiogenesis

PTEN is a tumor suppressor and genome keeper, its frequent mutation or deletion in cancer (57) is responsible for cell cycle deregulation and reprogramming (58, 59). PTEN deficiency and hypoxia synergistically condition the composition and organization of tumor microenvironment (60, 61). Hypoxic condition effectively maintains a low PTEN level and even slight changes in the doses of active PTEN cause deep effects in cancer. This is fundamental for the angiogenic process. Indeed, PTEN is the main regulator of vessel formation because it rules the cross-talk between DLL4 in the tip cells and NOTCH in the stalk cells, signaling for migration and growth respectively (14) as a function of the pO_2_ level (62).

As a tumor suppressor gene, PTEN is a tool to regulate the tumor microenvironment and a key target molecule for angiogenesis-based immunotherapeutic approaches. In fact, vessel normalization through PTEN activation in endothelial cells has several positive consequences. The first is pO_2_ increase, due to blood flow establishment, which increases the active form of endothelial PTEN. Secondly, targeting the active PTEN is a strategy to maintain vessels normalization, aiming to increase the efficacy of anticancer treatments including the new immunoregulating strategies (12).

## Impact of Hypoxia on the Antitumor Immune Response and Functional Consequences of Vascular Normalization

### Hypoxia Interferes With Antitumor Immune Response

The role of tumor hypoxia in the recruitment of immunosuppressive cells and the regulation of their functions is becoming evident. Tumor hypoxia impacts the antitumor immune response by promoting local immune suppression and inhibiting immune killing functions. In fact, hypoxic zones in solid tumors are infiltrated by a large number of the most widely studied immunosuppressive cells within the tumor microenvironment including myeloid derived stromal cells (MDSCs), tumor-associated macrophages (TAMs), and T-regulatory (Treg) cells (63). The hypoxia transcriptional factor HIF-1α has been directly shown to regulate the function and differentiation of MDSCs within the hypoxic TME. In this regard, we have provided evidence indicating that tumor-derived MDSCs are more immunosuppressive than splenic MDSCs mostly because of the induction of HIF-1α-dependent increased arginase activity and nitric oxide production. In addition, we showed that tumoral MDSC expression of PD-L1 is upregulated under hypoxia and increased MDSC-mediated T cell tolerance (64). In this respect, we, and others (65) further provided evidence that HIF-1α regulates the expression of PD-L1 by binding directly to a hypoxia response element in the PD-L1 proximal promoter.

TAMs have also been found to be preferentially located in tumor hypoxic areas, where they accumulate HIF-1 and HIF-2 (66). Furthermore, it has been reported that HIF-1 and HIF-2 play a role in the promotion of macrophage angiogenic properties (67, 68). It should be noted that dendritic cells (DCs) are also diverted by hypoxia from their highly specialized antigen-presenting and T cell-activating functions. In this regard, hypoxia has been reported to inhibit the stimulatory capacity of DCs for activating T cell functions (69). On the contrary, the production of proinflammatory cytokines, such as TNF and IL-1, as well as the inflammatory C-C chemokine receptor (CCR) type 5 (CCR5), was strongly upregulated under hypoxia in DCs (70). Moreover, several studies indicate that the maturation and function of DCs are influenced by several hypoxia-modulated factors, such as VEGF present in the tissue microenvironment. The production of VEGF-A by human tumors inhibits the functional maturation of DCs and, thereby, promotes immune escape of tumor cells (69). With respect to Tregs induction and immunosuppressive function, hypoxic stress has been reported to upregulate Foxp3 through direct binding of HIF-1 to the Foxp3 promoter region, inducing Treg cell formation (71). Facciabene reported that tumor hypoxia also attracts Treg cells inside the tumor bed by impacting the cytokine profile (72). Furthermore, hypoxic stress results in an increased expression and secretion of CCL28 by ovarian tumor cells, which acts as a chemoattractant for Treg cells. In a previous report, we have demonstrated that hypoxic stress, by inducing the pluripotency factor NANOG in tumor cells, activates the expression and secretion of the tumor immunosuppressive TGF-β1 by a mechanism involving at least direct binding of NANOG to the TGF-β1 promoter (73). Further, targeting this factor decreases TGF-β1 and reverses the intra-tumoral immune cell infiltrate by increasing the number of CD8+ T cells and decreasing the number of macrophages and Treg cells (73) in mice melanoma. These findings connect stem cell-associated factors with inhibition of the immune response in the hypoxic tumor environment.

It should be noted that the precise molecular mechanisms by which hypoxia alters the balance between growth-promoting inflammation and the antitumor immune response is not fully understood. Although the role of hypoxia in the induction of immunosuppression is unquestionable, it remains however complex and sometimes contradictory with respect to its role in promoting antitumor immunity. Very recently, HIF-1α expression was reported to correlate with increased tumor immune and stromal signatures and aggressive phenotypes in human cancer (74). Doedens et al. provided elegant data indicating that elevated HIF-1 and HIF-2 support the function of cytotoxic CD8+ T-cells (75). Tyrakis et al. have suggested a role for HIF-1α in CD8+ T-cell proliferation, differentiation, and antitumor activity through the regulation of L-2HG (76). In addition, HIF-1 was found to contribute to natural killer cell priming and activation *via* regulation of the glycolytic rate (77). Clearly more studies should be directed towards a better understanding of the precise molecular mechanisms by which hypoxia alters the balance between growth-promoting inflammation and the antitumor immune response.

### Barriers Posed by the Hypoxia-Angiogenesis Axis

Hypoxia in tumors is the first signal to proximal and distal endothelial cells (ECs) to undertake angiogenesis. It modulates the expression by endothelial cells of several adhesion molecules like E- and P-selectins, ICAM-1, VCAM, which are responsible for leukocyte trafficking (78–80). Thus hypoxia modifying the endothelial cell properties in the tumor, causes reduction of immune cell infiltration (81), maintains anergy of endothelial cells, their unresponsiveness to pro-inflammatory stimuli (82, 83), and reduced adhesion molecules levels (84). Both ICAM-1 and VCAM are low in EC in the tumors (85–87). Upon normalization of pathological angiogenesis, adhesion receptors are upregulated on ECs (88). ICAM-1 (89) or VCAM (90) are re-expressed and T cell infiltration into the tumor is also restored (91), as shown during the course of anti-VEGF therapy (92). Moreover, low migration of T cells into the tumor due to overexpression of the endothelin B receptor (ET(B)R) on ECs was reverted by ET(B)R blocking, which restored immune cell infiltration by ICAM-1 activity (93).

Another important endothelial regulator of anti-cancer immune response is PD-L1/PD-1 axis (94). During recruitment, CD8+ T cells binding to endothelial PD-L1 leads to downregulation of lymphocyte activation (95). Simultaneously, PD-L1 expressing ECs can support the suppressive functions of Tregs (96, 97). Given that PD-L1 is upregulated on tumor endothelial cells (94, 98), this contributes to make the pathogenic vessels a barrier for the development of a protective immune response in the tumor (99).

### Vascular Normalization Strategies vs Anti-Angiogenic Therapies

Vascular normalization strategy takes into consideration restoring the leaky, abnormal blood vessels that feed tumors. This aims to alleviate tumor hypoxia which would result in improvement of drug delivery, induction of an efficient anti-tumor immune response, and inhibition of cancer stem cells differentiation (100).

The often used antiangiogenic drugs are selective tyrosine kinase inhibitors (TKIs) that target pro-angiogenic receptors, such as endothelial growth factor receptors (VEGFR) family and Platelet-derived growth factor receptors (PDGFR) (101, 102). The use of TKIs significantly improved survival of patients suffering from certain types of cancers, such as renal cell carcinoma, hepatocellular carcinoma and colorectal carcinoma (101). In various types of treatment it was demonstrated that TKI’s can normalize vessels (103, 104). Moreover, treatment of breast cancer patients with bevacizumab induced the recruitment of pericytes to vessels, strengthening them in the primary tumor, and decreasing levels of circulating biomarkers VEGF, and Ang-2 (105), typical for hypoxia. This observation and knowledge that pericytes may express VEGFs as survival factor for neighboring ECs (106), led to the conclusion that more mature vessels may acquire independence from tumor-secreted VEGF-A, which may show resistance to bevacizumab (107). Altogether, vessel normalization has a huge positive impact on the tumor microenvironment, by increasing blood perfusion, alleviating tumor hypoxia, and reducing vessel leakiness resulting in increased vessel maturity. However, in such therapies the effects of vessel normalization were transient, and not effective for all types of cancer (100). To overcome the disadvantage of hypoxia-related limitations of antiangiogenic therapies, some approaches are emerging that emphasize stable normalization without excessive pruning of vessels and will be discussed (12).

As VEGF-A is the key inducer of angiogenesis, researchers designed a gene therapy based on a soluble form of the VEGF receptor-2 (sVEGFR2) (108–110) to trap and neutralize overproduced VEGFs. Such a strategy resulted in reduction of tumor growth and inhibition of pathological angiogenesis. Another strategy to normalize vessels was devoted to the oxygen sensing enzymes that regulate HIF-1α stability, namely the three prolyl hydroxylases (PHD1-3) enzymes (12, 111). Recently, several PHD inhibitors have been developed and evaluated in clinical trials (112, 113); studies on Lewis lung carcinoma showed that such inhibitor can normalize the TME, improving vessel maturation and reducing hypoxia (113).

A distinct approach to overcome hypoxia and normalize vessels is to use myo-inositol trispyrophosphate (ITPP), which is a membrane-permeant allosteric effector of hemoglobin (114). Human microvascular endothelial cells (MECs) (115), cultured in hypoxia in the presence of red blood cells (RBCs) pre-treated with ITPP, do not form “vessel like” structures as control cells. Moreover, markers of hypoxia such as HIF-1α and VEGF-A were dramatically reduced by O_2_ delivery ITPP-loaded RBCs upon rolling onto the endothelial cell layer in hypoxic conditions. Such results confirmed that ITPP enhances the capacity of Hb to release bound oxygen, which alleviates hypoxia and may be applied to reduce the extent of the pathological angiogenesis (116). Melanoma and breast cancer studies using ITPP-based treatment proved the vessels normalization properties of this molecule. Vessel maturation and strengthening were obtained, due to recruitment of pericytes and induction of VEGF-R2 in response to the pericyte/EC cross-talk. Maturation of vessels was also confirmed by upregulation of Tie-2 protein and upregulation of endothelial cell junctions. Long-term treatment with ITPP reduced PHD1-3, VHL, HIF-1, HIF-2, and HO-1 (tumor protective enzyme), which suggests stable reoxygenation of tumors (12). Moreover, ITPP treatment reduced selection of the cancer stem cell population and dissemination of cells from tumor, by downregulating CD133, MDR1, Oct-4 and Osteopontin. In addition, ITPP treatment led to a significant reduction in the number of cells expressing Glut-1, LDH and CAIX, showing that ITPP also regulates glycolysis in the TME (12).

Important elements of the TME that are involved in angiogenesis are endothelial precursor cells (EPCs), which include subpopulations of cells with different functional capacities. Recently, it has been shown that developed endothelial precursor cells (117) injected intravenously can be recruited to the tumor microenvironment site (109, 118). This process is mimicking the behavior of circulating progenitor cells during cancer development (109). Nowadays cell-based therapies are becoming more relevant (119, 120) and considering the homing properties of endothelial precursor cells (109, 121), it could be possible to engineer such early EPCs that would carry therapeutic genes and targeting angiogenesis (109, 122, 123). Collet et al. confirmed this hypothesis (118). Consequently, stable vessel normalization is confirmed to improve tumor microenvironment, in contrast to antiangiogenic therapy where normalization is only transient.

### Impact of Hypoxia on Transient vs Stable Normalization

As highlighted above, vessel normalization indeed modifies the tumor microenvironment, wherein the improved pericyte recruitment and upregulation of endothelial cell junctions allows for stable and mature vessels with decreased leakiness and proper perfusion (124). The key result of this phenomenon is reduction of hypoxia and pH increase in cancer microenvironment. Given that hypoxia induces selection of aggressive and metastatic cells, its reduction should inhibit the selection of cancer stem-like cells (CSCs). This was indeed observed in the tumor site following vessel normalization, as was the reduction in metastasis (12, 111).

There are several strategies that target vessel normalization, but only those which stabilize vessels constantly can give better therapeutic effects. As described in a previous section, an important role in stable vessel normalization is played by tumor suppressor PTEN in endothelial cells. This protein is one of the key regulators of proliferation of endothelial cells, it inhibits vessel sprouting and tube-like structures formation. PTEN was discovered to be very often mutated in cancer diseases (125). Overexpression of PTEN resulted in inhibition of angiogenesis and of tumor growth (126). This evidence suggested that targeting PTEN may be crucial in achieving stable vascular normalization. ITPP by its pyrophosphate structure was a good candidate to activate endothelial PTEN, which down regulates the PDK/PI3K/AKT/mTOR pathway and controls the angiogenesis process. This result was confirmed by downregulation of LOX protein which is involved in inhibiting PTEN expression (12).

## Potential Application of Hypoxia Biomarkers in Guiding Vascular Normalization Strategies

Stable vascular normalization strategies can be aided in their transition from the preclinical to the clinical setting through the availability of biomarkers for patient selection and treatment monitoring. A biomarker is defined based on the clinical question it addresses (7, 127). For vascular normalization, biomarkers that can delineate appropriate patient cohorts and that allow monitoring of response are essential for minimizing unwarranted side-effects and maximizing therapeutic efficacy. Hallmarks of vascular normalization entail improved vascular integrity through decreased permeability, and enhanced tumor perfusion, which alleviates tissue hypoxia (7, 127). Detecting changes in these phenomena has been accomplished through the determination of several imaging parameters and circulating plasma/serum markers and have recently been reviewed in the context of anti-angiogenic therapy (7, 127, 128). Herein, we discuss how combinational approaches considering the hypoxic state of the tumor as well as immune infiltration could prove impactful as surrogate biomarkers for the success of vascular normalization.

### Detection of Hypoxia in the Context of Vascular Normalization

Alleviation of hypoxia is a prolific end-result of an intact tumor vasculature and there are several methods by which this phenomenon can be detected in tumors, including immunohistochemical staining for intrinsic protein markers of hypoxia, notably CAIX, VEGFA, and HIF-1 (129, 130), as well as the extrinsic molecule pimonidazole (131). This reagent binds cellular macromolecules upon injection into patients, forming stable adducts with reduced proteins in conditions of low oxygen, that can then be detected by immunohistochemistry indicating hypoxic sites (131). Apart from these tissue-based markers, several imaging approaches have been designed for mapping tumor hypoxia in a non-invasive manner and some of these techniques have additionally been integrated to simultaneously monitor tumor vascular normalization.

Direct quantification of oxygen levels has been successfully achieved *in vivo* by applying both non-imaging electron paramagnetic resonance (EPR) oximetry and EPRI (imaging) approaches (132–134). Examination of tumor blood flow, prefusion or transient vascular normalization coincided with tumor oxygenation levels measured by these techniques in multiple pre-clinical cancer models (135–139). EPR oximetry requires the respective implantation of an exogenous particulate or the injection of a soluble agent into the tumor to quantify pO_2_ (132–134, 140). Detection is therefore limited to superficial tumors; however investigations to assess and put forth safer and more informative oxygen sensors that can work at any tissue depth are ongoing (133, 141–143), and some are being applied in clinical studies (144). Regarding EPRI, the recent introduction of nontoxic, water-soluble, and biocompatible paramagnetic probes could lead to its clinical translation, enabling the non-invasive, real-time quantification of pO_2_ in a three-dimensional format (134, 140, 145). Nonetheless, work remains to be done to alleviate safety concerns in human subjects, and to provide appropriate tools for human application (146).

Blood oxygenation level-dependent magnetic resonance (BOLD-MRI), which indicates oxygen saturation in the blood, and its counterpart, tissue oxygen level dependent (TOLD)-MRI, which measures local tissue oxygen concentration, have shown high potential as non-invasive qualitative indicators of tumor oxygenation (147). BOLD-MRI has recently been used in a colon cancer xenograft model treated with antiangiogenic agents; wherein an increase in the measured oxygen parameter, reflecting hypoxia relief, matched the transient vascular normalization, indicated by dynamic contrast enhanced magnetic resonance imaging (DCE-MRI) (148). DCE-MRI is a technique that has been applied in clinical trials for antiangiogenic therapy (127), as it enables monitoring tumor perfusion and vascular permeability (149). Vascular perfusion measured by DCE-MRI was verified to occur in an inverse manner to hypoxia in xenografts of pancreatic ductal carcinoma (150), patient-derived xenograft models of cervical cancer (151) and primary liver cancer (149). Such findings underscore the relevance of determining hypoxia reversal as a proxy indicator of mature and functional tumor vasculature.

Hypoxia can indirectly be assessed by applying computed tomography (CT) and positron emission computed tomography (PET) in combination with radiotracers. PET/CT has been applied with the radiolabeled glucose analogue ^18^F-fluorodeoxyglucose (FDG), which distinguishes cells in the TME based on their glucose metabolism, with preferential accumulation in hypoxic and slowly dividing cells (152). ^18^F-FDG PET/CT measures both hypoxia and metabolism and this technique has primarily been used in the clinic for tumor visualization, staging, restaging and monitoring treatment (152–154). Indeed due to its hypoxia selectivity, ^18^F-fluromisonidazole is the most widely used PET tracer for detecting tissue hypoxia in patient studies (130, 155). ^18^F-FMISO-PET relies on the entrapment of nitroimidazole compounds in hypoxic cells upon binding intracellular macromolecules (130), which allows for 3D visualization of intra-tumoral hypoxia. Preclinical studies have shown the utility of this technique in monitoring vascular normalization by highlighting the alleviation of hypoxia (12, 156). The application of ^18^F-FMISO-PET with other imaging modalities in glioblastoma showed that tumor hypoxic zones overlapped with hypervascularization (157, 158), however these vessels were characterized by abnormal permeability (159). This is consistent with the expected vascular integrity in hypoxia-driven angiogenesis (25). These studies further highlight the link between an abnormal tumor vasculature and hypoxia, reaffirming the utility of hypoxia indicators in monitoring vascular normalization

The interplay between hypoxia and angiogenesis is not a straightforward cause and effect relationship, as such spatial heterogeneity between hypoxic zones and degree of perfusion also exists in tumors (154, 160, 161). In particular, a pilot study on head and neck cancer patients that underwent ^18^F-FMISO dynamic PET (dPET), wherein hypoxia and perfusion were simultaneously determined at distinct time points, reported random trends between hypoxia and perfusion in the same tumor (160). In addition, a study on a small cohort of breast cancer patients that applied a multimodal imaging approach, revealed a negative association between ^18^F-FMISO-PET measured hypoxia and markers of vascular function and perfusion indicated by DCE-MRI; however there were areas that displayed one characteristic but not the other (161).

Taken together, for imaging methods to be applied to determine hypoxia relief as a proxy to vascular normalization, a multimodal approach that allows for extracting data on both phenomena might be the best route. Moreover, the reported relationship between hypoxia and vascularization is evidently tumor dependent, necessitating the testing and standardization of imaging parameters and thresholds as per cancer type.

### Relevance of Gene Signatures and Hypoxia Score in Defining a Tumor’s Hypoxic State

Hypoxia gene signatures have been derived to capture the multifaceted and heterogeneous transcriptional response to hypoxia across a range of solid tumors and have been nominated for potential clinical application as the next surrogate biomarker for hypoxia (15–18).

Hypoxia signatures have been used to classify patient tumors into high-hypoxia and low-hypoxia, based on a hypoxia score generated from the expression of the signature genes in tumor samples (16, 18). Alternatively, patients have been stratified according to a hypoxia risk score, which takes into consideration both the expression of each gene and the coefficient representative of its prognostic relevance (162–168). Despite the variety of methods used in both deriving and applying the signatures, the dichotomization of patient data based on hypoxia scores has generated strong prognostic power; wherein patients having deeply hypoxic tumors, experienced worse survival parameters [reviewed in (18)]. Moreover, at least four hypoxia signatures have shown predictive power of response to the addition of hypoxia modifying therapy to radiotherapy, or the addition of bevacizumab to neoadjuvant chemotherapy (**Table 1**). In all cases, the presence of hypoxia predicted improved response and when an interaction test with the treatment arm was performed, there was a significant difference in response to the combination therapy between the more hypoxic and less hypoxic tumors (**Table 1**). In addition, the increased ratio of expression of two hypoxia related genes was found to correlate with the absence of response to anti-PD1 in a small cohort of melanoma patients (174). Some of these are even being investigated in ongoing clinical intervention trials for head and neck (NCT01950689, NCT01880359, NCT02661152) and cervical (NCT04275713) cancers (175).

**Table 1:** Predictive hypoxia gene signatures.

Hypoxia signature	Patients^#^	Therapy^§^	Endpoint	Statistical test	Impact of hypoxia on combination therapy	Interaction between hypoxia and treatment arm
15-gene (169)	323 patients with head and neck squamous cell carcinoma from the DAHANCA 5 trial	Radiotherapy +/- CON	Locoregional failure	Multivariate Cox PH analysis	HR = 0.42; 95% CI: 0.25–0.68; P = 0.0001	P = 0.003
26-gene (170)	157 patients with T2–T4 laryngeal cancer from the ARCON trial	Radiotherapy +/- CON	Regional control	Univariate Cox PH analysis	HR* = 0.14; 95% CI: 0.03–0.62; P = 0.009	–
24-gene (171)	185 patients with locally advanced bladder carcinoma from the BCON trial	Radiotherapy +/- CON	Local relapse free survival	Univariate Cox PH analysis	HR = 0.47; 95% CI: 0.26-0.86; P = 0.015	P = 0.0094
28-gene (172)		Radiotherapy +/- CON	Overall survival	Univariate Cox PH analysis	HR = 0.54; 95% CI: 0.32–0.91; P = 0.021	P = 0.0026
3-gene (173)	289 patients with HER2-negative breast cancer from the GeparQuinto trial	Neoadjuvant chemotherapy +/- bevacizumab	Pathologic complete response	Multivariate logistic regression	OR = 2.40; 95% CI: 1.28–4.51; P = 0.006	P = 0.023

^#^Randomized phase III trials: DAHANCA 5, The Danish head and neck cancer; ARCON, accelerated radiotherapy with carbogen and nicotinamide; BCON, bladder carbogen nicotinamide; GeparQuinto: Bevacizumab, Everolimus (RAD001), and Lapatinib as Neoadjuvant Chemotherapy Regimes for Primary Breast Cancer.

^§^CON: carbogen and nicotinamide as hypoxia modifying therapy.

*Values taken from (16).

PH, proportional hazard; HR, hazard ratio; CI, confidence interval; OR, odds ratio.

Among the published signatures, a 15-gene signature (176) (**Table 2**) has been put forth as the best performer, showing high robustness in its ability to define a hypoxic state across different cancer types (177, 178). This signature has been applied to report on the molecular underpinnings (179) and mutational load (180) distinguishing high hypoxia and low hypoxia tumors in a pan-cancer setting. Moreover, it has been utilized to highlight distinct molecular features and their correlation with sensitivity to anticancer drugs in around 10,000 tumors classified based on their hypoxia status (178). Indeed, the application of hypoxia signatures in this manner highlights their utility in representing tumor hypoxia and their relevance in classifying patient tumors based on their hypoxic state.

**Table 2:** 15-gene Buffa hypoxia signature (176).

HGNC Symbol	Gene Name	Function
*ACOT7*	acyl-CoA thioesterase 7	Lipid metabolism
*ADM*	adrenomedullin	Angiogenesis
*ALDOA*	aldolase, fructose-bisphosphate A	Glucose metabolism
*CDKN3*	cyclin dependent kinase inhibitor 3	Cellular proliferation
*ENO1*	enolase 1	Glucose metabolism
*LDHA*	lactate dehydrogenase A	Glucose metabolism
*MIF*	macrophage migration inhibitory factor	Inflammation
*MRPS17*	mitochondrial ribosomal protein S17	Mitochondrial translation
*NDRG1*	N-myc downstream regulated 1	Stress response
*P4HA1*	prolyl 4-hydroxylase subunit alpha 1	ECM remodeling
*PGAM1*	phosphoglycerate mutase 1	Glucose metabolism
*SLC2A1*	solute carrier family 2 member 1	Glucose uptake
*TPI1*	triosephosphate isomerase 1	Glucose metabolism
*TUBB6*	tubulin beta 6 class V	Cytoskeleton organization
*VEGFA*	vascular endothelial growth factor	Angiogenesis

Recently, the genes themselves were interrogated through an integrative analysis that assessed the correlation between the expression of each of the 15 genes with their methylation status and copy number variations (CNVs), among other features, in pan-cancer data and in repositories of cancer cell lines (181). All genes presented with CNVs, and their expression levels were reflective of their decreased promoter methylation in most cancers. Of relevance, the genes were associated with increased activation of oncogenic signaling pathways, and enrichment of malignant behaviors reflective of hypoxia, invasion and EMT (181). Furthermore, they acted as risk factors in more than half of gene-cancer pairs, supporting their cancer promoting role (181). *VEGFA*, *SLC2A1*, and *LDHA* are three of these genes that play central roles in angiogenesis (*VEGFA*) and glucose metabolism (*SLC2A1* and *LDHA*) by encoding for VEGF, GLUT-1, and LDHA, respectively. As described earlier, these proteins are reduced post-ITPP vascular normalization (12); therefore the presence of such genes in hypoxia signatures underlines their potential to inform on the state of vascular normalization.

The concept of describing the hypoxic state of a tumor using a score that can determine a patient’s prognosis and guide their treatment trajectory is a highly attractive notion, however the published signatures require prospective clinical validation (16). Another consideration would be defining the cutoffs to be used for indicating the degree of tumor hypoxia, as well as understanding what control genes to integrate for quantifying gene expression and setting up the appropriate assays accordingly (16, 182, 183). Of relevance, a recent study compared the ability of distinct gene expression methodologies (qPCR, nCounter, and GeneChip) to score patients from two retrospective head and neck squamous cell carcinoma (HNSCC) cohorts of the DKTK-ROG (German Cancer Consortium Radiation Oncology Group), using three distinct hypoxia gene signatures, as well as other signatures (184). Based on expression data availability from the examined techniques, they reported on similar classification of patients as having more hypoxic or less hypoxic tumors. In addition, for the two signatures that had expression data from all three techniques, there was high concordance in the association of the scores from the signatures with disease locoregional control (184). This study underscores the robustness of gene panels in stratifying patients and their transferability across various quantification techniques (184).

It should be noted that hypoxia gene signatures have a measurable advantage due to their multiparametric nature. By integrating genes with varying hypoxia sensitivities and spatiotemporal dependency, they could buffer out discrepancies that would otherwise arise with single hypoxia indicators (183, 185). This has been shown in HNSCC (182, 185) and cervical cancer (183), where in comparison to single markers of hypoxia, multigene signatures showed stronger refractory power to intratumor heterogeneity. While such findings need to be confirmed for other cancer types as well, they support the notion that hypoxia scoring can be done using the same diagnostic tumor biopsy. This has an additional advantage of negating the need for radioactive contrast agents and tracers as those required by imaging.

A caveat to consider with hypoxia gene signatures is that they are primarily based on signaling pathways that are downstream of HIF-1, limiting their application in tumors harboring HIF activating mutations, including those with *VHL* mutations. Moreover, while they mark the presence of hypoxia, gene signatures do not give information on oxygenation levels. Despite these hurdles, a recent study on cervical cancer showed that combining a hypoxia gene signature with DCE-MRI, resulted in an improved and more informative evaluation of hypoxia related resistance to chemoradiotherapy (175). Therefore, it could be speculated that in vascular normalization strategies, hypoxia signatures could provide a supplementary datapoint about the TME that could prove valuable when applied alone or in conjunction with other imaging approaches, to assess the reversal of an abnormal tumor vasculature. Given that a normalized vasculature is expected to reprogram the tumor immune microenvironment as well, integrating immune markers could have an additional role in guiding approaches for vascular normalization.

### Combinational Transcriptomic Indicators of Hypoxia and Immunity

Assessment of the immune conditions of the tumor microenvironment has been carried out in glioma (163), hepatocellular carcinoma (HCC) (164), and lung adenocarcinoma (165), following their biphasic stratification into hypoxia high-risk and hypoxia low-risk groups (**Table 3**). Immune cell populations were determined and compared in the two groups using the analytical tool CIBERSORT (Cell-type Identification By Estimating Relative Subsets Of RNA Transcripts), which relies on the gene expression matrix of 547 genes to delineate 22 immune cell types in the TME (186). Distinct immune populations were shown to dominate in the high-risk and the low-risk groups in the tested cohorts (163–165) (**Table 3**). In particular, the high-risk groups in the HCC and GB datasets both showed higher prevalence of immune cells associated with an immunosuppressive TME, including Treg and M0 or M2 macrophages (163, 164). This was coupled by an increase of immune activating cell populations, NK cells, CD8+ T cells, and M1 macrophages, in the HCC hypoxia low-risk group (164) (**Table 3**). Further comparing the expression of immunosuppressive cytokines and immune checkpoints in GB, revealed their upregulation in the hypoxia high-risk group (163). On the other hand, the HCC hypoxia low-risk group showed increased immune score, based on ESTIMATE (Estimation of STromal and Immune cells in MAlignant Tumor tissues using Expression data) (187), which infers the stromal and immune cell fractions in a tumor sample using gene expression signatures. The increased immune score indicates higher infiltration of immune cells in regions of lower hypoxia (164). These findings are in line with reports from hypoxia-immune signatures, which include a mixture of hypoxia- and immune-related genes, and have similarly associated the presence of tumor hypoxia with an immunosuppressed state (188–190).

**Table 3:** Immune cell populations in tumors grouped based on hypoxia risk scores.

Immune cell population^#^	Comparison between hypoxia-high and hypoxia-low risk groups^§^					
	GB-High risk	HCC-High risk	LUAD-High risk	GB-Low risk	HCC-Low risk	LUAD-Low risk
B cell naïve	–	P = 0.266	P = 0.088	–		P = 0.51
B cell memory	–	P = 0.468		–		**P < 0.001**; **P = 0.025**
Plasma cells	–	P = 0.411	P = 0.515	–		P = 0.323
T cells CD8	–		P = 0.436	–	P = 0.072	P = 0.519
T cells CD4 naïve	–	–	P = 0.989; P = 0.38	–	–	
T cells CD4 memory resting	**P = 0.001**; **P = 0.001**				P = 0.082	**P < 0.001**; **P = 0.012**
T cells CD4 memory activated	–		**P < 0.001**; **P < 0.001**	–	P = 0.226	
T cells follicular helpers	–	P = 0.606	P = 0.857; P = 0.132	–		
T cells regulatory	**P = 0.006**; **P < 0.001**	**P = 0.077**				P = 0.265; P = 0.56
T cells gamma delta	–	P = 0.727	P = 0.384; P = 0.09	–		
NK cells resting	**P = 0.009**; **P < 0.001**	P = 0.992	**P < 0.001; P < 0.001**			
NK cells activated	–		P = 0.426; P = 0.672	**P < 0.001;** P = 0.683	**P = 0.004**	
Monocytes	–	P = 0.673		–		**P = 0.001**; **P = 0.001**
Macrophages M0	**P = 0.001**; **P = 0.03^ǂ^**	**P < 0.001**	**P < 0.001; P < 0.001**			
Macrophages M1			**P < 0.001; P = 0.02**		**P = 0.011**	
Macrophages M2					P = 0.526	P = 0.845; **P = 0.04**
Dendritic cells resting	–	P = 0.856		–		**P < 0.001**; **P < 0.001**
Dendritic cells activated	–	P = 0.134	P = 0.308	–		P = 0.193
Mast cells resting	–			–	**P < 0.002**	**P < 0.001**; **P < 0.001**
Mast cells activated	–	P = 0.081	**P < 0.001**; **P = 0.013**	–		
Eosinophils	–	P = 0.093	P = 0.409	–		P = 0.489
Neutrophils	**P < 0.001**; **P = 0.001**	**P = 0.002**	**P = 0.035**			P = 0.167

**^#^**Expression of the immune cell populations was derived based on the analytical tools CIBERSORT (Cell-type Identification By Estimating Relative Subsets Of RNA Transcripts) (163–165).

**^§^**Hypoxia-high and hypoxia-low risk groups were compared in glioma (GB) (163), hepatocellular carcinoma (HC) (164) and lung adenocarcinoma (LUAD) datasets (165). P-values for the increasing immune populations in each group are presented. Two P-values are presented for GB and LUAD, which included two independent datasets. P-values < 0.05 were considered statistically significant and are presented in bold.

**^ǂ^**Increase was reported for tumor associated macrophages in total (163).

- data has not been reported in the paper.

Prospective studies are required to validate the clinical impact of these signatures. Nonetheless, they provide proof of concept for the application of signatures to embody distinct facets of the TME including hypoxia and immune activation and predict patients’ clinical outcomes. These features can be harnessed in the context of vascular normalization wherein signatures could act as indirect indicators for the reversal of an abnormal vasculature. Alternatively, the signatures could play a role in predicting treatment trajectories when vascular normalization approaches are used in combination with other treatment modalities, like immunotherapy. This concept has recently been applied to generate a mathematical model to predict impact of vascular normalization on the success of immunotherapy (9). Considering markers of vascular integrity and mechanical tumor properties (9) and advancing combinational approaches that integrate TME parameters, should realize the translational impact of vascular normalization strategies.

## Discussion

Immunotherapeutic strategies are witnessing an explosion and constitute a disruptive revolution in cancer treatment thanks to their significant efficacy and lasting benefits (191). However, despite the significant progress, we are currently facing a wide range of challenges. There is still a need for more effective treatments to maximize cancer patient survival rates. A challenge related to the hostility and complexity of the tumor microenvironment remains to be further elucidated to position immunotherapy as a transformative and the most used approach for the treatment of cancer. Clearly a conflict between the tumor system and an unfavorable tumor microenvironment able to neutralize or paralyze the immune system of the host exists. Several TME associated cellular and metabolic factors contribute to the failures in cancer therapies, including immunotherapy (192). In this regard, hypoxia, which is a hallmark of solid tumors, plays a crucial role in tumor promotion and immune escape by conferring tumor resistance, immunosuppression, and tumor heterogeneity. Hypoxia in solid tumors also promotes angiogenesis that is required for tumor progression as tumor cell growth frequently outstrips the supply of oxygen and nutrients. The HIF-1 mediated transcriptional response to hypoxia is cell-type specific and involves an orchestrated expression of angiogenic growth factors by various cell types within the hypoxic tissue in a temporally and spatially regulated manner. Several angiogenic factors including in particular VEGF-A bind to their cognate receptors on target cells within the stroma and regulate their behavior and reactivity to the immune cells. They also participate to the recruitment of immune cells, forming the immune infiltrate to fight the tumor cells but which, under the influence of the hypoxia-dependent composition of the TME, are changing their phenotype or are selected to help the tumor cells development and dissemination (193). Angiogenesis and its functional state, together with the TME operate in a crosstalk to control the possibility of treatments and to rule the type and quality of the immune response. It appears thus, as a necessity to focus on the search for means to control it. Normalization is indeed a concept that has, and will, change the combined treatment approaches. To get a better understanding of the means that one can apply to patients, the large scale gene analysis taking into account hypoxia appears necessary under the conditions that it is combined with the immune-related interpretation of the data and pooling the various new and old parameters helping to understand the TME vs tumor cells reactions (194).

At such point the biological and biochemical implications of the angiogenesis-based mechanisms and the possibility they offer to control drug accessibility to tumor cells on the one hand as well as the TME composition and cellular reactions on the other hand make their modulation fundamental. This opens to new roads for the immune checkpoints control. PD1/PD-L1 interactions, among immune checkpoints give an example of the huge potential provided by the angiogenesis normalization approaches as the poor effects of the treatments obtained to date imply that those strategies should be revisited in the hypoxia alleviation context (4).

## Author Contributions

RA, KB, AF, CK, and SC contributed to writing the manuscript. NZ, SB, and CS contributed to revising the manuscript. All authors contributed to the article and approved the submitted version.

## Funding

CK and AF were supported by Ministry of Defense grant Kosciuszko I no 579/2016/DA. CK was supported by National Scientific Center grants no OPUS2016/23/B/NZ6/02542 and OPUS2016/23/B/NZ1/03211. KB was supported by Military Institute of Medicine intramural grant no 1/8910 (414).

## Conflict of Interest

The authors declare that the research was conducted in the absence of any commercial or financial relationships that could be construed as a potential conflict of interest.
